# Influence of Chronic Stroke on Functional Arm Reaching: Quantifying Deficits in the Ipsilesional Upper Extremity

**DOI:** 10.1155/2019/5182310

**Published:** 2019-02-26

**Authors:** Savitha Subramaniam, Rini Varghese, Tanvi Bhatt

**Affiliations:** Department of Physical Therapy, University of Illinois at Chicago, Chicago, IL 60612, USA

## Abstract

**Purpose:**

The purpose of this study was to quantify ipsilesional upper extremity (UE) stand-reaching performance (kinematics and kinetics) among chronic stroke survivors.

**Method:**

Community-dwelling chronic stroke survivors (n=13) and age-similar healthy adults (n=13) performed flexion- and abduction-reaching tasks. Surface EMG and acceleration were sampled using wireless sensors from the prime movers (anterior and middle deltoid) and provided performance-outcome (reaction time, burst duration, movement time, and movement initiation time) and performance-production (peak acceleration) measures and were then evaluated.

**Results:**

Individuals with chronic stroke demonstrated significantly reduced performance outcomes (i.e., longer reaction time, burst duration, movement time, and movement initiation time) and performance production ability (i.e., smaller peak acceleration) compared to their healthy counterparts (*p* < 0.05) for both flexion- and abduction-reaching movements.

**Conclusion:**

Our results are suggestive of post-stroke deficits in ipsilesional motor execution during a stand-reaching task. Based on these findings, it is essential to integrate ipsilesional UE training into rehabilitation interventions as this might aid functional reaching activities of daily living and could ultimately help community-dwelling chronic stroke survivors maintain their independent living.

## 1. Introduction

On average, only 5% of chronic stroke survivors regain complete function of their contra-lesioned upper extremity (UE), with 20-80% of survivors having persistent motor deficits [1, 2]. Contra-lesional deficits increase individuals' reliance on their ipsilesional UE for activities of daily living and maintaining functional independence. However, in the past decade, studies have indicated the presence of motor deficits in the ipsilesional UE among individuals with chronic stroke [3–6]. Laboratory-based, kinematic studies have demonstrated ipsilesional upper and lower extremity deficits during the performance of various motor tasks. Such deficits were seen substantially in the finger tapping task (lesser mean number of taps in five 10 second trials), goal-directed aiming task (decreased peak acceleration, final target position error, and increased time to complete the task), and cyclical single-limb movements (cycle durations were higher, and movement amplitudes were lesser) of the ipsilesional upper and/or lower extremity among people with chronic stroke [7, 8].

Another recent large cohort study evaluated the effect of subacute stroke on ipsilesional UE motor deficits during a visually guided seated-reaching task using the KINARM robotic exoskeleton [9]. Based on the study results, the authors suggested that kinematic measurements of ipsilesional UE were similar to those reported in previous studies, such as initial direction error, along with increased reaction time and movement time. Additionally, based on the current research on ipsilesional UE motor deficits, recent studies are exploring and developing rehabilitation methodologies to improve gross motor function. A pilot study exhibited substantial improvements in ipsilesional UE movement kinematics (reduction in distance error, direction error, and aspect ratio) and functional performance (Jebsen-Taylor Hand Function Test, Fugl-Meyer assessment, and Functional Independence Measure) during a seated target reaching task, for a three-week (1.5 hours/session) training paradigm that included practice of real-life ipsilesional UE tasks in a virtual-reality environment [10].

While the current research emphasis is on tasks suitable to understand and rehabilitate ipsilesional UE control and deficits, the majority of studies have only focused on UE target reaching from a seated position. Despite the fact that seated UE reaching is relevant to workplace-related activities, stand-reaching functional tasks are comparatively more challenging, owing to the preparatory demands of the movements, including postural muscle preparation and maintaining the center of mass (CoM) within the base of support (BoS) [11]. Forward and lateral UE functional reaching activities are used abundantly in the real world, and muscles of the shoulder complex such as the anterior and middle deltoid play a principal role in these movements [12]. Additionally, very few studies have reported the use of a stand-reaching task among individuals with chronic stroke [13, 14], and among those that did make use of this task, the majority focused on examining lower extremity postural responses and did not consider activation of the UE muscles.

In addition to UE stand-reaching functional task performance, rapid arm movements facilitate the generation of anticipatory feed forward mechanisms during self-induced perturbations [11, 15]. Such mechanisms promote recovery of equilibrium (postural stability), by regulating the relationship between the body's CoM and the BoS. Studies have also indicated that a rapid muscle force generated at the shoulder joints can facilitate a higher degree of stabilization as reaching with an UE to grasp or touch an object for support rapidly changing the BoS, thus generating “change-in-support” reactions [16]. Given that stand reaching ability is an essential prerequisite to UE functional task performance and is also necessary for independent living and preventing falls via rapid arm movements, it is critical to evaluate ipsilesional UE motor function in standing among community-dwelling individuals with chronic stroke.

Quantification of ipsilesional UE motor deficits in individuals with chronic stroke using functional ability measures (Functional Independence Measure, Chedoke-McMaster Stroke Assessment, and Purdue Pegboard) has found minimal differences compared to healthy controls [9]. Studies have used electromyography (EMG) for assessment of UE dysfunction and have demonstrated the use of both performance-production and performance-outcome measures to understand motor behavior in association with its neural control [17, 18]. Performance production measures may provide more insight into aspects of the nervous system during the performance of a motor task. In other words, the central nervous system (CNS) representation of movement control could be evaluated through performance-production measures (i.e., peak acceleration) [19, 20], and the result of a motor task performance could be evaluated using performance-outcome measures (i.e., reaction time, movement time, burst duration, and movement initiation time). Given the importance of the ipsilesional UE for functional task performance, as well as it being the primary contributor of ‘UE change-in-support' reactions to prevent falls, it is crucial to understand movement deficits of the ipsilesional UE in chronic stroke survivors.

Thus, the purpose of this study was to quantify the reaching performance of chronic stroke survivors' ipsilesional UE using both, outcome-based and production-based measures for a stand-reaching task, and then compare their performance to age-similar healthy adults. We hypothesized that, on both flexion- and abduction-reaching tasks, the ipsilesional UE of stroke survivors would show measurable deficits in movement control marked by significant deterioration in both performance- and production-based variables compared with age-similar healthy adults.

## 2. Methods

### 2.1. Participants

Twenty-six individuals participated in this study (N = 26). The study consisted of two groups: individuals with chronic hemiparetic stroke (62.75 ± 6.12, n = 13) and age-similar healthy adults (63.82 ± 6.34, n = 13). The ipsilesional arm was non-dominant for n = 8 and dominant for n = 5 participants. The Institutional Review Board for the University of Illinois at Chicago approved the study, and informed consent was obtained from all the participants.

#### 2.1.1. Subject Eligibility

Inclusion criteria for stroke survivors consisted of the physician's confirmation of chronic hemiparetic stroke. Participants with a history of a single cortical stroke, confirmed by their physician, were also recruited for this study. Individuals were required to be able to stand independently for at least five minutes without the use of an assistive device so that they would be able to complete the functional arm reach task. Participants' mean ± SD disability status was quantified using the Modified Rankin Scale, and the participants ranged from mild to moderate disability (2.72 ± 0.49) [21]. Both stroke and healthy adult participants answered a general health questionnaire, filled out a Physical Activity Scale for the Elderly (PASE) [22], and demonstrated a comfortable and full range of motion for the shoulder. Participants were excluded upon reporting recent surgeries (< 6 months). Participants with active signs or symptoms of any discomfort of the shoulder and any other neurological (e.g., Parkinson's disease, vascular (multi-infarct) Parkinsonism, vestibular deficits, peripheral neuropathy, or unstable epilepsy), musculoskeletal, and cardiovascular disorders and cognitive deficits (score > 10 on Short-Orientation-Memory-Concentration (SOMC) test of Cognitive Impairment) were also excluded [23]. Individuals with cardiovascular diseases as assessed by resting heart rate (> 85% of age-predicted maximal) and resting oxygen saturation (< 95) were also excluded.

### 2.2. Apparatus Design

**Figure 1:**
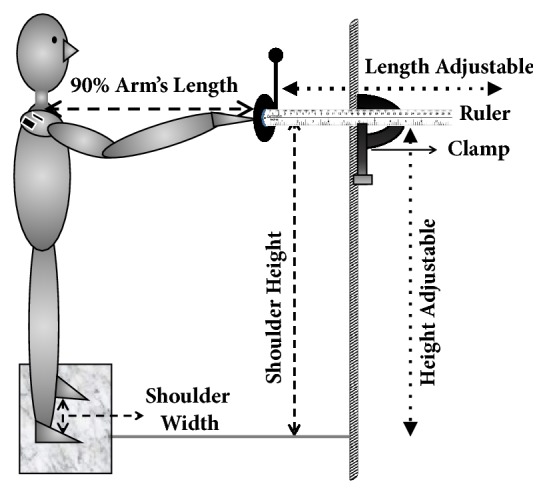
Experimental set-up showing custom-made apparatus, including a ruler and eye-fixator marker held by clamp complex, attached to the stationary pole and adjustable in height and length. Subject stood with shoulder width distance between feet, marked on a paper foot mat, reaching out to the target set at 90% of arm length. Trunk movement was controlled by instructing the subject to keep the shoulder blades in contact with the wall at all times. EMG sensors were affixed to the anterior and middle deltoid muscles of the dominant arm.

Set-up for the functional arm reach task included a custom-made arm reaching apparatus, which consisted of a long metal pole attached to a wooden board on the ground. A large load-bearing clamp was fixed to the metal pole, with the long-shaft of the clamp perpendicular to the length of the pole. The large load-bearing clamp was adjusted to participants' shoulder height. Another movable, smaller clamp was used to attach a 36 inch wooden ruler perpendicular to the shaft of the larger clamp. Circular foam of 4 inches in diameter with a 1 inch smaller target marked centrally was attached to one end of the wooden ruler. The target was adjusted in such a way that, it was kept at 90% of the participant's arm length. A passive marker, in line with the target, was taped to the top-end of the screw of the small clamp at a fixed distance of 3.5 inches to serve as gaze fixation. Delsys® Trigno™ wireless electromyography sensors were used to measure the electrical activity of the muscle. These sensors also had tri-axial accelerometers embedded in them that sampled the rate of change of velocity. Reliability analyses for the response variables used in this study have been comprehensively detailed previously [19]. The surface EMG and acceleration data sampled from the wireless sensors in the above study to assess flexion- and abduction-stand-reaching task performance yielded intertrial and test-retest reliability for both performance-outcome and performance-production measures. Such sensitivity and reliability are critical for measuring ipsilesional UE function among individuals with chronic stroke and healthy older adults. A schematic representation of the reaching set-up used in the study is shown in Figure 1.

### 2.3. Protocol and Instrumentation

Participant's footprints were marked on a paper mat while they were standing with a shoulder-width base of support. For the duration of testing, they were asked to stand within the markings of their feet, to maintain a constant base of support. For flexion reaching, participants stood to face the target and, for abduction reaching, the participants stood to face sideways with their arms at their sides. The target was set by adjusting the ruler to 90% of the participant's maximum arm length, defined as the distance from the acromion to the tip of the middle finger. Following three familiarization trials, participants were provided with three trials each of verbally cued forward reaching through shoulder flexion and sideways reaching through shoulder abduction. Once ready, Delsys EMGworks® Software generated automated verbal cues consisting of a first cue (preparatory), “Get Ready,” given at 2 seconds, where participants had to focus their attention at the passive marker visually, followed with a second cue, “Go,” given at 4 seconds, at which participants were required to reach out and touch the target “as quickly and as accurately” as possible and return to the starting position. Participants were instructed to keep their back supported against the wall between trials. If participants missed the target or started to do the reaching task before the second verbal cue was provided, that trial was repeated to ensure the precise performance of the task. All participants received a mandatory break of 2-3 minutes after a set of three trials to avoid fatigue. EMG signals from a total of six trials per participant were collected from the involved anterior and middle deltoid (i.e., the anterior deltoid for the three flexion-reaching trials and the middle deltoid for the three abduction-reaching trials) and then the variables of interest were extracted and analyzed, using a customized MATLAB code.

Delsys® Trigno™ surface electromyography (EMG) sensors were used to record EMG activity in the ipsilesional UE (anterior deltoid for shoulder flexion and middle deltoid for shoulder abduction) of individuals with chronic stroke and age-similar healthy adults. The sensors were placed in line with the muscle belly using hypodermal tape [24]. EMG signals were sampled at 2,000 Hz and were then hardware band-pass filtered over a bandwidth of 20–450 Hz, applying a standard mode rejection ratio of > 80 db. The sensors enclosed tri-axial accelerometers that sampled signals at 148.1 Hz over a bandwidth of 50 Hz and amplitude range of ±1.5 g. The EMG data was smoothed via digital high-pass filtering using a fourth-order zero-lag Butterworth filter with a cut-off frequency of 20 Hz. This was followed by full-wave rectification and a low-pass filter with a cut-off frequency of 50 Hz. This EMG signal was then used to obtain onset and offset latencies for the muscles (defined below). The acceleration signals were smoothed using a fourth-order low-pass Butterworth filter with a cut-off frequency of 80 Hz, and this signal was used for computing onset and offset to calculate movement initiation time and movement time. EMG signals from a total of six trials per participant were analyzed from the involved prime mover (i.e., the anterior deltoid for the three flexion-reaching trials and the middle deltoid for the three abduction-reaching trials) using a customized MATLAB code to extract the variables of interest.

### 2.4. Outcome Measures

#### 2.4.1. Primary Measures

*Performance-Outcome Measures*. Reaction time is the time needed to initiate a movement response following a visual, auditory, or other sensory signal and reflects the speed of transmission of the central nervous system. In this study, reaction time was the time interval calculated between the final auditory cue (“GO” at 4 s) and the initiation of EMG activity. Onset was identified as the time after four seconds when the EMG signal exceeded two standard deviations (SDs) of the ensemble average of the resting baseline EMG over one-second period (i.e., from 3 s to 4 s). Reaction time was measured in milliseconds (ms). Burst duration was defined as the total duration of EMG activity. It was calculated as the time elapsed between onset and offset latency of the EMG signal and was also measured in milliseconds. The offset was identified as the time point always occurring after the onset time point when the signal fell below 2 SDs of the initial resting baseline. Movement time was defined as the total time interval from the start to the end of upper extremity (UE) movement, taken as the interval between the onset (> 2 + SDs) and offset of acceleration (< -2 S. Ds). Movement initiation time is the time measured between the onset of EMG and the onset of acceleration signals, measured in milliseconds.

**Figure 2:**
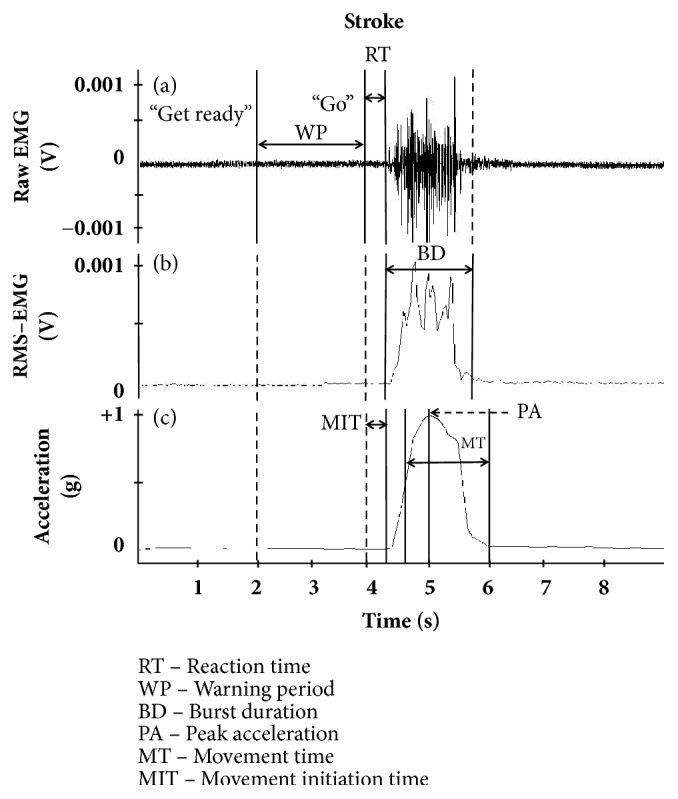
(a) Raw and (b) processed (filtered and rectified) EMG and (c) acceleration, sampled from the anterior deltoid during flexion-reaching, depicting variables of interest. Reaction time (RT) is the times between the final cue and the EMG onset. Burst duration is the time between start and end of EMG signal. Movement time (MT) was defined as the total time interval from the start to the end of the upper extremity movement, taken as the interval between the onset and offset of the acceleration signal. Peak acceleration (PA) was defined as the maximum amplitude of the acceleration signal along the X-axis of the sensor's coordinate system. Movement initiation time (MIT) is the time after RT to the beginning of the acceleration signal.

*Performance-Production Measures*. Peak acceleration was defined as the maximum amplitude of the acceleration signal along the X-axis of the sensor's coordinate system. We used the X-axis because the sensor was placed closer to the joint rather than to the endpoint of the shaft. Hence, movements occurred along the sagittal axis of the sensor. The unit of measurement was g, (1g = 9.8m/s/s). Raw and processed EMG signals and filtered acceleration trace from the anterior deltoid muscle during a single flexion-reaching trial demonstrate the performance-outcome measures, and performance-production measure is represented in Figure 2.

#### 2.4.2. Secondary Measures

To assess health status post-stroke, the Stroke Impact Scale (SIS) was administered using an interview that measures changes in eight impairment categories (strength, hand function, activities of daily living, mobility, communication, emotion, memory, and thinking) [25]. Participants were administered a short cognitive ability test composed of six items on the Short Orientation-Memory-Concentration (SOMC) test. The SOMC test has been previously validated as a measure of cognitive impairment, assessing changes in verbal memory. The test is scored on a scale from 0 to 28, with scores above 20 being representative of ‘normal cognition', and scores below 10 being indicative of ‘normal-minimal impairment' [23]. Participants were also evaluated on the falls-efficacy scale, which uses a 10-item questionnaire to assess one's confidence in their ability to perform ten daily tasks without falling as an indicator of their fear of falling impacting physical performance. Lower scores indicate more confidence and higher scores entail a lack of confidence and greater fear of falling.

### 2.5. Statistical Analysis

From the 26 participants, one stroke survivor who volunteered to participate in the study was excluded from analyses because of technical problems during data collection and another was excluded from the analyses due to signal contamination (noise), probably resulting from loose sensor contact. All remaining data (78 trials-39 trials of flexion and 39 trials of abduction), was analyzed. A mixed model ANOVA was conducted with independent variables being, group (age-matched and stroke), task (flexion and abduction) and trials (1-3), and dependent variables being reaction time, burst duration, movement time, movement initiation time, and peak acceleration. Significant interactions were resolved with post hoc paired t-tests for within-subject factors and independent t-tests for between-subject factors. Independent t-tests were performed to compare SOMC test scores between the groups. Age, weight, and height were compared between the stroke survivors and age-similar healthy adults using independent* t*-tests. A chi-square test was used to compare the sex distributions of the groups. Spearman's correlation was run to assess the relationship between UE functional reaching reaction time (ms) and falls-efficacy scale score in chronic stroke survivors. To assess if limb dominance confounded differences in burst duration between the stroke and the healthy controls; an independent t-test was used to compare burst duration between participants with concordant (dominant UE is stroke affected) and discordant (non-dominant UE is affected) strokes. A significance level (*α*) of 0.05 was chosen for all statistical comparisons. Analyses were performed using version 17.0 of the commercially available Statistical Package for the Social Sciences (SPSS).

## 3. Results

Both groups were similar in demographics (age, weight, height, and sex) (Table 1). The mean score for SIS hand function was 33.41 (S.D ± 5.56), indicating moderate impairment among chronic stroke survivors [26]. Furthermore, both groups exhibited moderate cognitive ability with a mean score of 5.21 (S.D ± 2.13) on the SOMC test.

**Figure 3:**
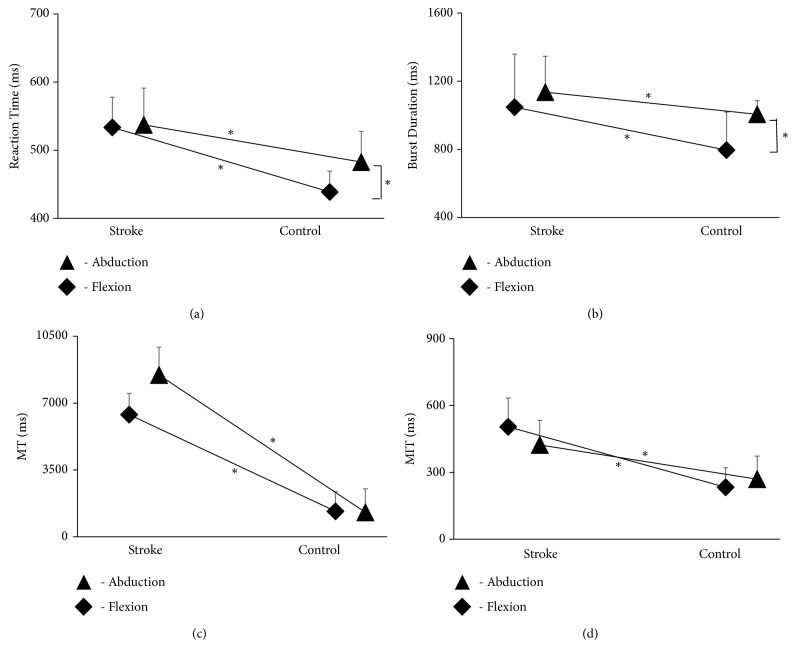
The data shows the effect of stroke on ipsilesional upper extremity in performance outcomes on (a) reaction time (ms), (b) burst duration (ms), and (c) movement time (MT) (ms) and (d) movement initiation time (MIT) (ms) compared to their healthy counterparts (p < 0.05) for both flexion- and abduction-reaching movements.

For reaction time, there was a significant main effect of task [F (1,48) = 2.003; p < 0.05] and group [F (1,24) = 14.555; p < 0.001], as well as a significant task × group interaction [F (1,48) = 1.492; p < 0.05]. However, there was no significant task × trial interaction [F (2,48) = 0.330; p =0.721]. There was also no significant task × group × trial interaction [F (2,48) = 0.081; p =0.922]. Post hoc analyses showed no significant difference in reaction time between flexion and abduction for the stroke group (p =0.695). However, reaction time for abduction was significantly greater than for flexion for the age-similar healthy adults (p < 0.05). There was also a significant increase in reaction time for the stroke group compared with the age-similar healthy controls for both flexion (p < 0.001) and abduction (p < 0.001) (Figure 3(a)).

For the burst duration, there was a significant main effect of task [F(1,48) = 19.127; (p < 0.001], and group [F(1,24) = 49.379; p < 0.001], as well as a significant task × group interaction [F(1,24) = 12.232; p < 0.001]. However, there was no significant task × trial interaction [F(2,48) = 2.022; p = 0.144]. Similarly, there was no significant task × group × trial interaction [F(2,48) = 1.670; p = 0.199]. Post hoc analyses showed no significant difference in burst duration between flexion and abduction for the stroke group (p = 0.87); however burst duration was significantly higher for abduction than for flexion (p < 0.001) for the age-similar healthy adults. Burst duration was also significantly greater for the stroke group compared to the age-similar healthy adults group for both flexion (p < 0.001) and abduction (p < 0.05) (Figure 3(b)).

For movement time, there was a significant main effect of task [F(1,48) = 159.543; p < 0.001], and there was a significant task × group interaction [F(1,48) =8.110; p < 0.05]. However, there was no significant task × trial interaction [F(2,48) = 1.076; p =.349)] and task × group × trial interaction [F(2,48) = 1.771; p = 0.181)]. Post hoc analyses exhibited no significant difference in movement time between flexion and abduction for the stroke group (p = 0.972) or for the age-similar healthy adults group (p = 0.167). However, the age-similar healthy adults group did demonstrate significantly decreased movement time compared to the stroke group for both flexion (p < 0.05) and abduction (p < 0.05) (Figure 3(c)).

For movement initiation time, there was no significant main effect of task [F(1,48) = 0.208; p = 0.652]; however, there was a main effect of group [F(1,24) = 14.844; p < 0.001]. There was no significant task × group interaction [F(1,48) = 1.426; p = 0.244)]. Similarly, there was no significant task × trial interaction [F(2,48) = 3.067; p = 0.056)] and task × group × trial interaction [F(2,48) = 0.714; p =0.495)]. Post hoc analysis showed significantly decreased movement initiation time in the age-similar healthy adults group compared to the stroke group for both flexion (p < 0.05) and abduction (p < 0.05) (Figure 3(d)).

**Figure 4:**
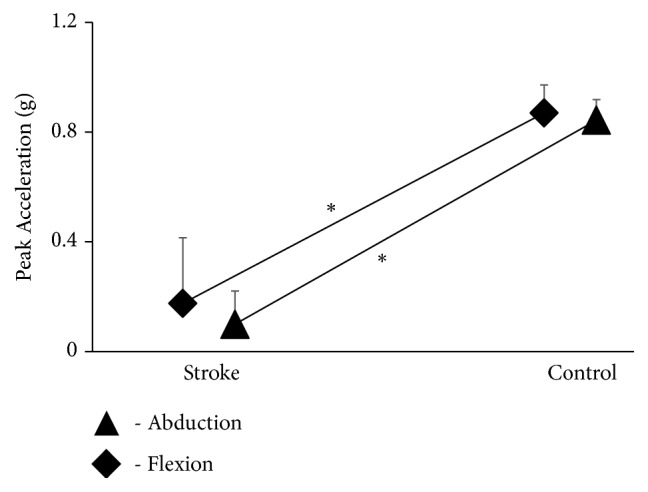
The data shows the impact of stroke on ipsilesional upper extremity in performance production outcomes on a) peak acceleration (g) compared to their healthy counterparts (p < 0.05) for both flexion- and abduction-reaching movements.

For peak acceleration, there was no significant main effect of task [F(1,48) = 1.004; p = 0.326]; however, there was a main effect of group [F(1,24) = 1643.784; p < 0.001]. There was additionally no significant task × group interaction [F(1,48) = 0.262; p = 0.613], task × trial interaction [F(2,48) = 0.382; p = 0.685] and task × group × trial interaction [F(2,48) = 0.873; p = 0.424]. The stroke group also exhibited reduced peak acceleration in comparison to age-similar healthy adults for both flexion (p < 0.05) and abduction (p < 0.05) (Figure 4).

Correlation analysis demonstrated a significant positive correlation between UE reaching reactions and Falls-self-efficacy scores (r_s_ = 72, p < 0.001). For flexion, burst duration was longer for participants whose ipsilesional arm was non-dominant (n = 8) than for participants whose ipsilesional UE was dominant (n = 5) for flexion; however, this difference was not statistically significant.

## 4. Discussion

Recognizing the impact of stroke on the ipsilesional upper extremity is an essential step for implementing effective rehabilitation. The purpose of this study was to quantify ipsilesional upper extremity reaching performance among chronic stroke survivors using a reliable stand-reaching paradigm simulating real-life functional reaching that has associated postural demands. This would then be used to compare them to age-similar healthy adults. Our findings indicate that chronic stroke survivors exhibited more severe deficits in movement control compared to age-similar adults, seen by significantly poorer performance-outcome and performance-production measures during the performance of a stand reaching task.

Individuals with stroke exhibited increased reaction time compared with age-similar healthy adults. Although the functional, multiplanar stand-reaching task has not yet been evaluated, other studies in stroke survivors also showed a disproportional delay in reaction time of the ipsilesional upper extremity in comparison to the control group [27]. Such increased response time in hemiparetic chronic stroke survivors has been indicated to be among the leading predictors for a decline in movement initiation, motor function performance, and functional outcomes [28, 29]. Further, the study found that reaction time was strongly correlated with falls self-efficacy. The significant associations between these variables could suggest that initiation of upper extremity motor response may play an essential role in the initiation and control of quick, protective grasping arm raising responses required to counter destabilization. Previous evidence has demonstrated that delayed choice reaction time on a lower extremity stepping task could significantly predict fall-risk among older adults [30]. More importantly, evidence has suggested that upper extremity finger-press choice reaction time has been used to distinguish between fallers and non-fallers [31].

The longer burst duration seen among stroke survivors in comparison to their counterparts may be attributed to their reduced ability to produce optimal levels of neuromuscular activation [32]. With these longer burst durations, it has been shown that ipsilesional deficits tend to be more profound when present on the nondominant side [10]. In line with previous research, this study also indicated increased burst duration when the ipsilesional side was the nondominant side (ipsilesional side as nondominant n = 8, ipsilesional side as the dominant n = 5). The delay in reaction time and the prolonged burst duration seen in the ipsilesional upper extremity could, therefore, be due to the lack of control mediated through the small percentage of descending cortical tracts that originate from the lesioned site in the dominant hemisphere [33].

Another very interesting finding from the current study was that the age-similar healthy adults presented with increased reaction time and burst duration for abduction, rather than for flexion, reaching tasks. Such differences could have resulted from the comparatively increased use of flexion-reaching rather than abduction-reaching during activities of daily living in age-similar healthy adults [34]. However, the above scenario of increased reaction time and burst duration for abduction compared to flexion-reaching tasks was not seen among the individuals with stroke. It could be postulated that the loss of shoulder joint flexibility contributed to the reduction of both flexion- and abduction-stand reaching task performance after stroke. Based on the current study results it is recommended that rehabilitation of ipsilesional upper extremity should include multidimensional (flexion and abduction) movement control training. However, more in-depth research is needed to understand and compare movement control for both flexion- and abduction- reaching tasks.

Similar to the results seen in the current study, previous work has demonstrated increased movement time of the ipsilesional upper-extremity in adults with unilateral brain damage during tasks such as Fitts' task [35], single target tapping [36], and sequencing hand postures [37]. Studies have suggested that this slowing of tasks could be due to the task requirement of rapid alternating changes in direction, difficulties in sequencing, and prolonged dwell time (i.e., time on target) for adults with chronic stroke [38, 39]. Visuospatial integration has also been shown to be associated with the high endpoint precision required for goal-directed task performance [40]. Furthermore, it has been demonstrated that poststroke individuals exhibited abnormal interactions between sensory systems (i.e., somatosensory, visual, and vestibular afferents), as well as impaired dynamic regulation of the integrated signals (sensory reweighing) of the ipsilesional UE [41]. Thus, this study, in line with others, adds to the research literature showing that the organization and execution of multisegment ipsilesional upper extremity movements are disrupted among chronic stroke survivors, ultimately resulting in slower task performance.

Prior studies using electromyography in stroke survivors have also demonstrated a significant delay in movement initiation time, similar to that seen in this study. Studies have indicated that such delay in simple functional tasks of the ipsilesional upper extremity can be associated with lesions causing specific impairments in motor processing and descending efferent mechanisms [38]. In general, the posterior parietal cortex and premotor areas mediate motor processing, while the primary motor and premotor areas mediate selection of motor strategy and motor execution [42]. Nonetheless, studies have indicated that the eventual motor output among stroke survivors is modulated by changes in descending and propriospinal excitatory and inhibitory inputs into the spinal interneurons and alpha motoneurons [43]. However, the degree to which this difference was due to impairment in motor processing rather than the impairment of efferent mechanisms would need further investigation.

Compared to age-similar nondisabled healthy adults, chronic stroke survivors demonstrated lower peak acceleration for both flexion- and abduction-reaching tasks. These findings are consistent with previous literature supporting the presence of kinematic deficits in movement velocity and acceleration, on the ipsilesional side [3]. These deficits have been postulated to indicate poor anticipatory planning, an essential component of motor skills [44, 45]. Such deficits in movement planning could be attributed to the disruption of ipsilateral contributions from the affected hemisphere. Bilateral corticospinal contributions to each arm are crucial for economizing the speed of control processes [46]. Specifically, ipsilateral corticospinal processing time is slower than crossed contralateral tracts and may be especially important for early movement planning (inhibitory influence of the ipsilateral motor cortex on responses to stimulation of the human cortex and pyramidal tract). Similarly, transcallosal exchanges of information are slower than ipsilateral hemisphere processing alone [47, 48]. Thus, the lack of ipsilateral control after a stroke can have a significant impact on the speed of movement and hence can impact the amplitude of peak acceleration. Alternatively, lower peak accelerations could have been due to impaired regulation of force and firing pattern of single motor units on the ipsilesional upper extremity [49, 50]. Although there is a lack of concrete evidence to support this hypothesis, it serves as a credible avenue for further research.

Several possible mechanisms could account for the ipsilesional motor deficits among chronic stroke survivors. A dominant theory suggests that the ipsilateral uncrossed descending corticospinal pathways may play an important role in the movement control of the ipsilesional upper extremity [46, 48]. A stroke event causes injury to the uncrossed corticospinal system [51] thus impairing such supplementary ipsilateral control. An alternate hypothesis is that ipsilesional movement control depends on complex interhemispheric communication between cortical (i.e., dorsal premotor cortex, supplementary motor area) areas likely mediated through the corpus callosum for the interhemispheric transfer of perceptual, sensory, and motor information underlying complex and integrated behaviors [42].

While it is well known that the ipsilesional upper extremity in people with chronic stroke exhibits significant motor deficits which lead to reduced accuracy and efficiency of movements [5, 36], the majority of evidence in this area is from seated upper extremity movement tasks. In the current study, we have focused on extending this evidence to include reaching while standing. Our findings concur with the previous studies and additionally allow us to conclude that ipsilesional deficits persist even in standing. This is particularly of note because depending upon the stroke severity the ipsilesional upper extremity may play a dominant role in maintaining or restoring stability control during full-body tasks, such as walking [52]. To best understand the role of the upper extremity standing balance and falls, the study adopted a reaching task that imposed similar demands on response programming as would be evident during standing [16, 53]. This is crucial because, as indicated earlier, upper extremity responses a change-in-support reactions during standing appear to be important for maintaining standing balance and recovering from perturbations [54, 55].

The findings from this study add to existing literature by relating ipsilesional motor deficits of upper extremity to falls self-efficacy among stroke survivors. Future research is required to investigate the characteristics of the ipsilesional upper extremity during recovery from a fall. The current research does, however, suggest that the implementation of training paradigms to improve motor control in the ipsilesional UE may be crucial for the rehabilitation of chronic stroke survivors.

## Figures and Tables

**Table 1:** Demographics and stroke characteristics of community-dwelling stroke participants are shown in the table.

Subject	Sex M/F	Age (year)	Weight (kg)	Height (cm)	I Side (L/R)	Concordant/ Discordant	Type (H/I)	Onset (year)	SIS (UE)	SIS (LE)	SOMC	FES
n = 13	5/8				7/6	8/5	6/7					

Mean		60.75	93.48	169.27				9.72	33.41	31.45	5.21	15.36

SD		5.12	41.27	8.80				3.32	5.56	7.36	2.13	2.76

I Side = Involved Side, M = Male, F = Female, L = Left; R = Right; H = Hemorrhagic; I = Ischemic; M = Male; F = Female; BMI = Body Mass Index; Concordant stroke = Dominant hand is also stroke-affected hand or Discordant = Dominant hand is not stroke-affected), SIS = Stroke Impact Scale, UE = Upper Extremity, LE = Lower Extremity, SOMC = Short Orientation-Memory-Concentration Test of Cognitive Impairment, and FES = Falls-Efficacy Scale.

## Data Availability

The data used to support the findings of this study are included within the article.

## Abbreviations

UE: Upper extremity
ADL: Activities of daily living
PASE: Physical Activity Scale for the Elderly
SOMC: Short-Orientation-Memory-Concentration
EMG: Electromyography
RT: Reaction time
MT: Movement time
MIT: Movement initiation time
BD: Burst duration
PA: Peak acceleration
N: Number
DS: Dominant side
AS: Affected side
L: Left
R: Right
H: Hemorrhagic
I: Ischemic
M: Male
F: Female
BMI: Body mass index
FES: Falls-Efficacy Scale
LE: Lower extremity
SIS: Stroke Impact Scale.

## Additional Points

*Highlights*. (i) Ipsilesional UE stand reaching is critical to understand anticipatory feed-forward mechanisms. (ii) Poststroke individuals exhibited deficits in movement control during a stand reaching task. (iii) Interventions should include ipsilesional UE training to improve motor control and prevent falls.
